# Upper brainstem cholinergic neurons project to ascending and descending circuits

**DOI:** 10.1186/s12915-023-01625-y

**Published:** 2023-06-06

**Authors:** Peilin Zhao, Tao Jiang, Huading Wang, Xueyan Jia, Anan Li, Hui Gong, Xiangning Li

**Affiliations:** 1 Britton Chance Center for Biomedical Photonics, Wuhan National Laboratory for Optoelectronics, MoE Key Laboratory for Biomedical Photonics, Huazhong University of Science and Technology, Wuhan, 430074 China; 2 Institute of neurological diseases, North Sichuan Medical University, Nanchong, 637100 China; 3 Research Unit of Multimodal Cross Scale Neural Signal Detection and Imaging, Chinese Academy of Medical Sciences, HUST-Suzhou Institute for Brainsmatics, JITRI, Suzhou, 215123 China; 4 Key Laboratory of Biomedical Engineering of Hainan Province, School of Biomedical Engineering, Hainan University, Haikou, 570228 China

**Keywords:** Projection patterns, Cholinergic neurons, Pedunculopontine nucleus, Laterodorsal tegmental nucleus, Single cell, Morphology

## Abstract

**Background:**

Based on their anatomical location, rostral projections of nuclei are classified as ascending circuits, while caudal projections are classified as descending circuits. Upper brainstem neurons participate in complex information processing and specific sub-populations preferentially project to participating ascending or descending circuits. Cholinergic neurons in the upper brainstem have extensive collateralizations in both ascending and descending circuits; however, their single-cell projection patterns remain unclear because of the lack of comprehensive characterization of individual neurons.

**Results:**

By combining fluorescent micro-optical sectional tomography with sparse labeling, we acquired a high-resolution whole-brain dataset of pontine-tegmental cholinergic neurons (PTCNs) and reconstructed their detailed morphology using semi-automatic reconstruction methods. As the main source of acetylcholine in some subcortical areas, individual PTCNs had abundant axons with lengths up to 60 cm and 5000 terminals and innervated multiple brain regions from the spinal cord to the cortex in both hemispheres. Based on various collaterals in the ascending and descending circuits, individual PTCNs were grouped into four subtypes. The morphology of cholinergic neurons in the pedunculopontine nucleus was more divergent, whereas the laterodorsal tegmental nucleus neurons contained richer axonal branches and dendrites. In the ascending circuits, individual PTCNs innervated the thalamus in three different patterns and projected to the cortex via two separate pathways. Moreover, PTCNs targeting the ventral tegmental area and substantia nigra had abundant collaterals in the pontine reticular nuclei, and these two circuits contributed oppositely to locomotion.

**Conclusions:**

Our results suggest that individual PTCNs have abundant axons, and most project to various collaterals in the ascending and descending circuits simultaneously. They target regions with multiple patterns, such as the thalamus and cortex. These results provide a detailed organizational characterization of cholinergic neurons to understand the connexional logic of the upper brainstem.

**Supplementary Information:**

The online version contains supplementary material available at 10.1186/s12915-023-01625-y.

## Background

The central nervous system has a highly ordered structure: external information is transmitted to a higher center through an ascending pathway, and decision-making information is delivered via a descending pathway to determine an individual’s physiological changes and behavior [1]. The upper brainstem (midbrain and pons), located between the diencephalon and medulla, has dense interconnections with different regions of the nervous system, from the spinal cord to the cortex [2, 3]. Previous studies have indicated that specialized populations of these neurons preferentially innervate the ascending and descending circuits. Dopamine (DA) neurons in the ventral tegmental area (VTA) [4] and different types of neurons in the dorsal raphe (DR) [5–7] send most of their axons to ascending circuits and a few to descending circuits. Two separate groups of glutamatergic neurons in the mesencephalic locomotor region (MLR) participate in different functions by projecting to the ascending and descending circuits, respectively [8]. Furthermore, cholinergic neurons in the pontine-tegmental cholinergic system, including the pedunculopontine nucleus (PPN) and laterodorsal tegmental nucleus (LDT), send abundant axons in both the ascending and descending circuits [9]; however, their projection patterns at the single-cell level have not been systematically characterized until now.

As the main cholinergic system in the rostral hindbrain [10], pontine-tegmental cholinergic neurons (PTCNs) send abundant axons along three major trajectories and are involved in various functions [11]. In the ascending dorsal circuits, PTCNs mediate prefrontal serotonin release from the DR [12] and participate in multiple functions, including auditory sensation [13], sensorimotor function [14], and spatial memory [15], by targeting different thalamic nuclei. Additionally, PTCNs innervate different neurons in the striatum (STR) and contribute to exploratory motor behaviors and action strategies [16]. In the ascending ventral circuits, previous studies have mainly focused on cholinergic modulation of the VTA and substantia nigra (SN), which are critically involved in mechanisms of reward [17], addiction [18], locomotion [19] and depressive-like behaviors [20]. In descending circuits, PTCNs govern the activities of the pontine reticular nucleus and contribute to various functions, including muscle tone suppression, inhibition of ongoing movement [11], and mediation of the prepulse inhibition of startle [21]. They also modulate breathing by projecting to the retrotrapezoid nucleus [22] and even to skeletal muscles via polysynaptic pathways [23].

There are approximately 2000 PTCNs in one hemisphere of the mouse brain [24]. How does the pontine-tegmental cholinergic system innervate many brain regions and contribute to various functions with a limited number of neurons? Notably, the activation of PPN cholinergic neurons plays an opposite role in locomotion in the ascending and descending circuits [11]; however, the relationship between these two circuits is unclear. Although previous studies have acquired whole-brain projections of PTCNs [9], it is still unclear whether the ascending and descending axons belong to different groups or to the same group of neurons, which is urgently needed at the single-neuron level. Previous studies [25] have reconstructed partial axons in serial slices and provided preliminary evidence that individual PPN neurons have complex axonal projections. However, we lack the unabridged single-cell connection of the pontine-tegmental cholinergic system due to limited techniques for tracing and imaging.

In this study, we combined sparsely labeled fluorescence micro-optical sectioning tomography (fMOST) serial technologies [26, 27], and acquired a morphological atlas that may have uncovered the projection logic of PTCNs at the single-cell level.

## Results

### Sparse labeling and single-cell reconstruction

**Fig. 1:**
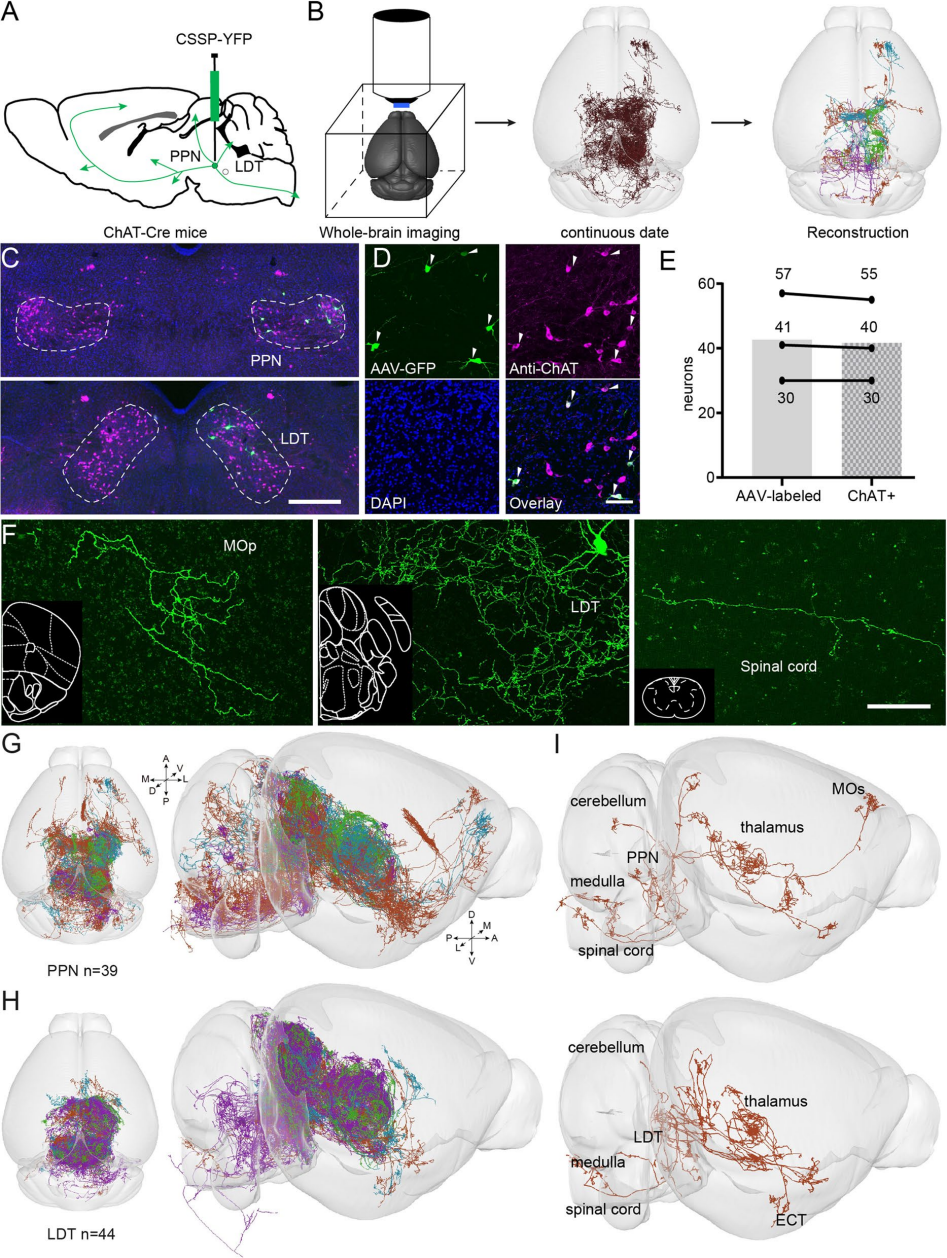
Reconstructions of the individual cholinergic neurons. **A** Diagram of the sparse labeling with the virus. **B** The main steps for whole-brain data acquisition and single-cell reconstruction. **C** Sparsely labeled neurons in the PPN and LDT. **D** A four-panel presentation with AAV-YFP, anti-ChAT, DAPI, and merged, arrows point out cholinergic positive neurons. **E** Calculation of all labeled neurons and ChAT+ labeled neurons (*n* = 3 mice). **F** A clear view of Labeled axons in different regions, such as the cortical area, inject site, and spinal cord. Scale bar = 100 μm. **G**, **H** Reconstructed neurons mapped to the Allen CCFv3 in 3D. PPN, *n* = 39 neurons; LDT, *n* = 44 neurons. Left is the plan view of reconstructed neurons. The right is the side view of reconstructed neurons in the whole brain. 83 neurons were reconstructed from six brains. **I** Single neurons stretched their axons to the cortex, thalamus, cerebellum, medulla, and spinal cord simultaneously

To obtain the fine morphology of individual cholinergic neurons, we employed a Cre-dependent virus for sparse labeling [28], the fMOST system for whole-brain imaging [26, 27], and GTree software for single-cell reconstruction [29] (Fig. 1A, B). First, we administered 100 ml CSSP-YFP to the PPN or LDT of ChAT-Cre mice. Four weeks later, the infected mice were sacrificed, and the target regions examined (Fig. 1C). Immunofluorescence staining of the slices verified the cell types of the labeled neurons (Fig. 1D, E). More than 96% of them were ChAT-positive (from three mice). These results indicate that the virus used had good specificity.

Subsequently, we embedded whole-brain samples with resin and acquired continuous datasets with a resolution of 0.32 μm × 0.32 μm × 1 μm via fMOST. Briefly, we fixed the sample on the base and acquired an image of the top surface using two fluorescent channels; the imaged tissue was subsequently removed. Thus, we obtained a continuous whole-brain dataset, layer by layer, with high resolution [26, 27]. To verify the quality of the datasets for single-cell reconstruction, we evaluated the labeled signals in the entire brain (Additional file 1: Fig. S1). We examined the labeled axons in different targets (Fig. 1F; Additional file 1: Fig. S1 D-Q) and found that the labeled signal had a good signal-to-noise ratio with the background, and single axons could be distinguished from each other regardless of whether the regions contained dense or sparse axons.

By combining continuous datasets with semiautomatic reconstruction methods [29], we reconstructed 83 assumed cholinergic neurons (PPN, 39 neurons; LDT, 44 neurons) in the pontine-tegmental cholinergic system (Fig. 1G and H; Additional file 1: Fig. S2). The reconstructed neurons had abundant collaterals throughout the brain, and some simultaneously projected to the cortex, cerebellum, and medulla (F ig. 1I). The axons of the reconstructed neurons covered the main targets of PTCNs [9] and could represent the morphological characterization of individual cholinergic neurons in the pontine-tegmental cholinergic system.

### The whole-brain projection logic of individual PTCNs

**Fig. 2:**
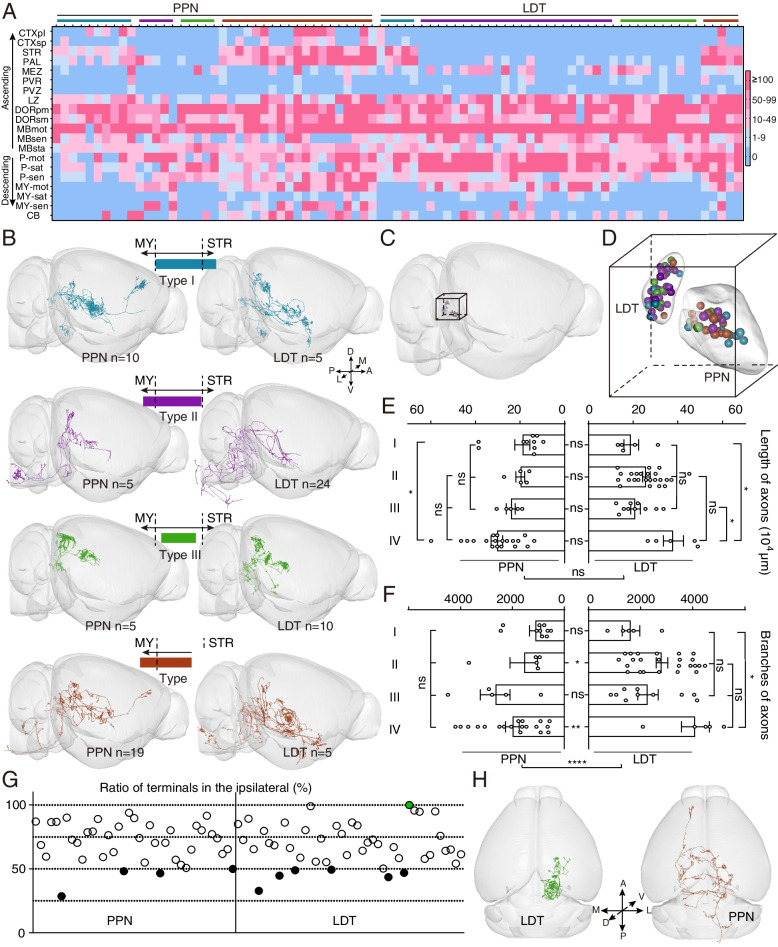
The whole-brain projection patterns of PTINS. **A** The distribution of axonal terminals of single neurons. Each column displayed one reconstructed neuron. Boxes in different colors explained the number of terminals of a single neuron in different brain regions. Different colors on the top represent reconstructed neurons that were classified into different types based on axons. **B** 3D view of typical neurons with different projection patterns. **C** The soma of all reconstructed neurons in the whole-brain outline. **D** A clear view of reconstructed neurons. The somas in the PPN and LDT neurons were isolated. The somas of neurons with different projection patterns were mixed. **E** Quantification and comparison of axon length of reconstructed neurons in four types. **F** Quantification and comparison of axon branches of reconstructed neurons in four types. **G** The ratio of axonal terminals in the ipsilateral of single neurons. The green dot showed a neuron confined its axons in the ipsilateral. Black-filled dots represent 10 neurons that had richer axons in the contralateral areas. **H** Left was the top view of the ipsilateral restricted neuron. The right was top the view of a typical contralateral preference neuron. Data are shown as mean ± SEM. Among four projections patterns, one-way ANOVA was followed by Tukey’s post hoc tests. Between the PPN and LDT, two-sided *t*-tests. *P < 0.05, **P < 0.01, ****P < 0.0001. Details of abbreviations for brain regions are shown in Nomenclature and abbreviations. For detailed statistics of axons, see Additional file 2: Table 1

To understand the projection patterns of individual PTCNs, we registered the reconstructed neurons to the Allen Mouse Brain Common Coordinate Framework version 3 (Allen CCFv3) [30] and quantified the terminals in the target regions (Fig. 2A). Similar to previous studies [11], we divided the targeted regions into two circuits: the midbrain and rostral regions belonging to the ascending circuits, and the pons, medulla, and spinal cord were classified as descending circuits. As shown in Fig. 2A, all reconstructed neurons had extensive collateralization in multiple areas, and most projected to various nuclei in the ascending and descending circuits simultaneously, except for two in the PPN. Moreover, we found that most reconstructed neurons projected to the thalamus, indicating that the thalamus is the major target of both the PPN and LDT (PPN, 38/39 neurons; LDT, 43/44 neurons). Our results showed that nearly half of the cholinergic neurons projected to the cerebellum (PPN, 19/39 neurons; LDT, 20/44 neurons), and some extended to the paraflocculus (PFL) (Additional file 1: Fig. S3A).

We noted that most individual PTCNs extend many axons to the three major demonstrated trajectories [11] simultaneously; thus, we could not analyze single-cell projection patterns with traditionally defined trajectories. Given that most reconstructed neurons project to the diencephalon, midbrain, and pons concurrently, previous studies on PTCNs have mainly focused on targets between the STR [16] and medulla [22]. To further investigate the projection patterns of PTCNs, we classified the reconstructed neurons into different groups and analyzed their characteristics. Four types were distinguished depending on whether their collaterals ascended to the STR or descended to the medulla (Fig. 2A, B). As shown in Fig. 2A and B, type I neurons targeted the anterior telencephalon with collaterals in the STR, but not in the medulla (PPN, 10 neurons; LDT, 5 neurons). Type II neurons preferentially projected to the posterior brainstem, including the medulla and spinal cord, but not to the STR (PPN, 5 neurons; LDT, 24 neurons). Type III neurons projected axons between the STR and medulla (PPN: 5 neurons; LDT: 10 neurons). Type IV neurons had the widest axons in both the STR and medulla (PPN, 19 neurons; LDT, five neurons). The PPN sent more cholinergic axons to ascending targets (type I and IV, PPN 29/39 neurons; LDT 10/44 neurons), whereas the LDT preferred the descending circuits (type II and IV, PPN 24/39 neurons; LDT 29/44 neurons). Furthermore, PPN neurons sent more divergent axons than LDT neurons (type IV: PPN, 19/39 neurons; LDT, 5/44 neurons). We investigated the location of the somas (Fig. 2C, D) and found that the cell bodies of neurons with different projection patterns in the same nucleus were intermingled (Additional file 1: Fig. S3B), indicating that adjacent PTCNs might have distinct projections. This agrees with the results of cholinergic neurons in the basal forebrain [24] and serotonergic neurons in the raphe [7].

To determine possible differences between neuronal populations with distinct projection patterns, we counted the lengths and branches of the reconstructed axons (Fig. 2E, F). We found that both the PPN and LDT neurons had abundant axons with lengths ranging from 9 to 60 cm (Fig. 2E). There was no significant difference in the length of axons between the PPN and LDT neurons. In the PPN group, type I neurons had shorter axons than type IV neurons (PPN, I vs. IV, *P* = 0.035). Among the four types of LDT neurons, type IV had the largest number of axons (LDT, IV vs. I, *P* = 0.0289; IV vs. III, *P* = 0.021). As the distributions of axons and synaptic connections do not always correlate [31], we compared the terminals of the reconstructed neurons. As shown in Fig. 2F, individual PPN and LDT neurons had abundant terminals ranging from 500 to 5200 and LDT neurons had richer terminals in total (PPN vs. LDT, *P* < 0.0001) or in different projection patterns (II, *P* = 0.029; IV, *P* = 0.0018). Among the different types, the number of terminals showed no significant difference in the PPN group, whereas type IV neurons in the LDT group had richer branches than type I neurons (LDT IV vs. I, *P* = 0.0156).

From a whole-brain perspective, most PTCNs extended their axons to both hemispheres. To investigate the projection patterns in the bilateral hemispheres, we counted the terminals in the targeted regions of single neurons and quantified the ratio of ipsilateral axons. As shown in Fig. 2G, most neurons projected to bilateral regions, except for one in the LDT, which confined its axons to ipsilateral areas. We also found several neurons (PPN, 4 neurons; LDT, 6 neurons; Additional file 1: Fig. S2), preferred the contralateral hemisphere and sent richer axons (Fig. 2H).

**Fig. 3:**
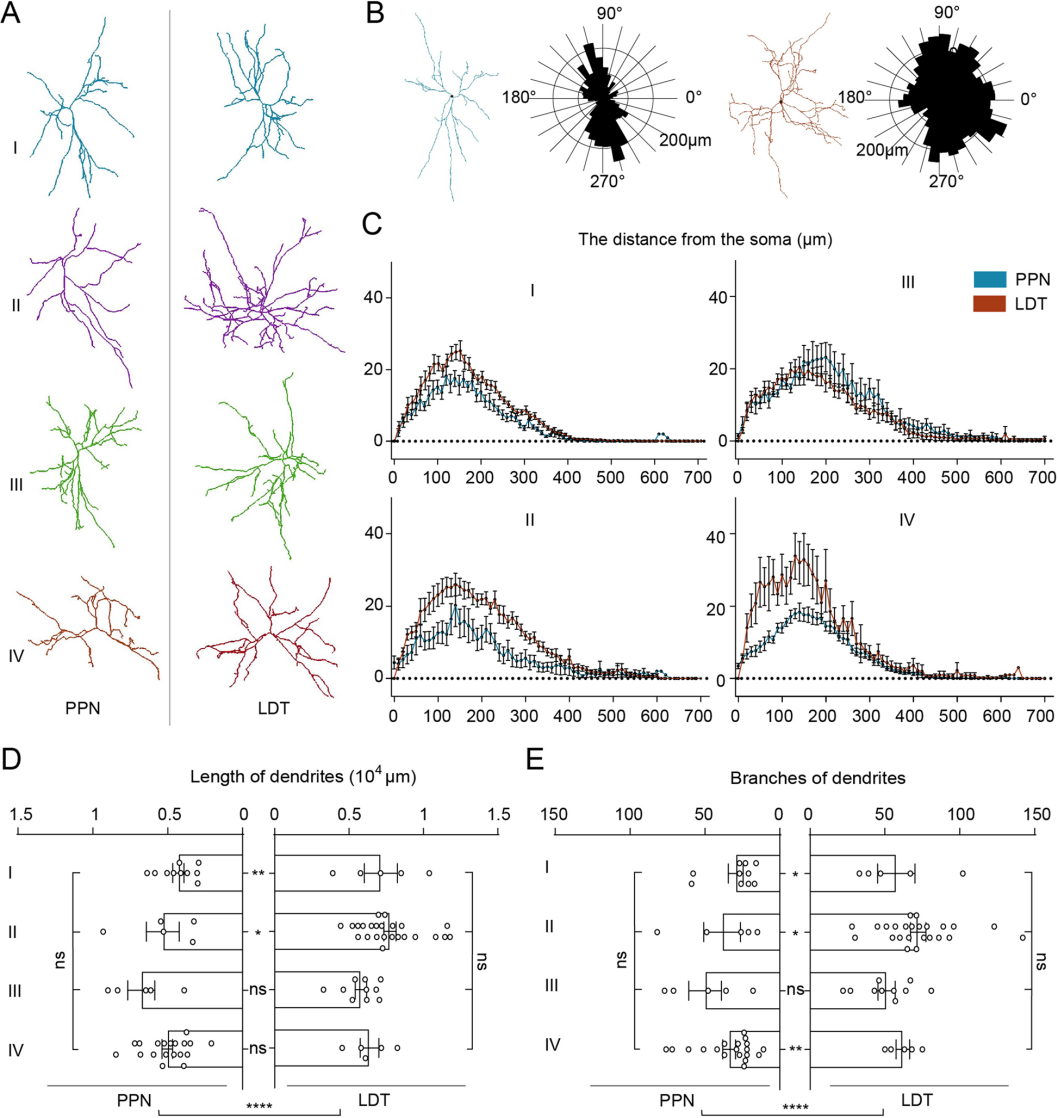
The dendritic morphology of PTCNs. **A** The typical dendrite of PTCNs in different projection patterns. **B** Polar analysis of dendrite. Left was the dendritic tree of a cholinergic neuron with a bipolar dendritic tree. The right was the dendritic tree of a cholinergic neuron with a multipolar dendritic tree. **C** Sholl analyses of dendrites of PTCNs in different projection patterns. **D**, **E** Quantification and comparison of length and branches of reconstructed dendrites. Data are shown as mean ± SEM. Among four projections patterns, one-way ANOVA was followed by Tukey’s post hoc tests. Between the PPN and LDT, two-sided *t*-tests. *P < 0.05, **P < 0.01, ****P < 0.0001. For detailed statistics of dendrites, see Additional file 2: Table 1

Dendrites are the portals through which neurons receive information from others. We found that the different projection patterns of PTCNs in the PPN and LDT had abundant dendrites (Fig. 3B). Consistent with previous studies [32], we divided the dendrites into bipolar and multipolar dendritic trees according to the distribution of longer dendrites (Fig. 3B) and found that a few had bipolar dendritic trees, whereas most were multipolar (Additional file 1: Fig. S4). We then analyzed the spatial distribution of dendritic branches using Sholl analysis (Fig. 3C). Our results suggested that the dendrites of both PPN and LDT neurons were mainly distributed about 600 μm away from the soma and most dendrites gathered in a 50-350 μm radius and the LDT neurons had richer dendrites. To confirm this, we quantified the lengths and branches of all the reconstructed dendrites. As shown in Fig. 3D and E, LDT neurons had significantly longer dendrites and more branches in total (*P* < 0.0001) in some subtypes, such as the length of type I (*P* = 0.0093) and type II (*P* = 0.0273), and the branches of type I (*P* = 0.0252), type II (*P* = 0.0129), and type V (*P* = 0.004) neurons. In the same nucleus, different types of neurons have similar dendrites and branches. This suggests that different types of PTCNs have similar dendrites, but LDT cholinergic neurons have richer dendrites than PPN neurons.

In summary, the single axon morphologies reconstructed here in the whole brain demonstrate that individual PTCNs send abundant collaterals to multiple targets in both hemispheres with various projection patterns. PPN neurons project more divergent axons to ascending and descending circuits, whereas LDT neurons contain richer axonal branches and dendrites.

### The projection logic of PTCNs in distinct circuits

Among the ascending circuits, the thalamus is one of the primary targets of the pontine-tegmental cholinergic system for information dissemination. PTCNs are involved in auditory sensations [13], sensorimotor actions [14], and spatial memory [15] by innervating different thalamic nuclei.

**Fig. 4:**
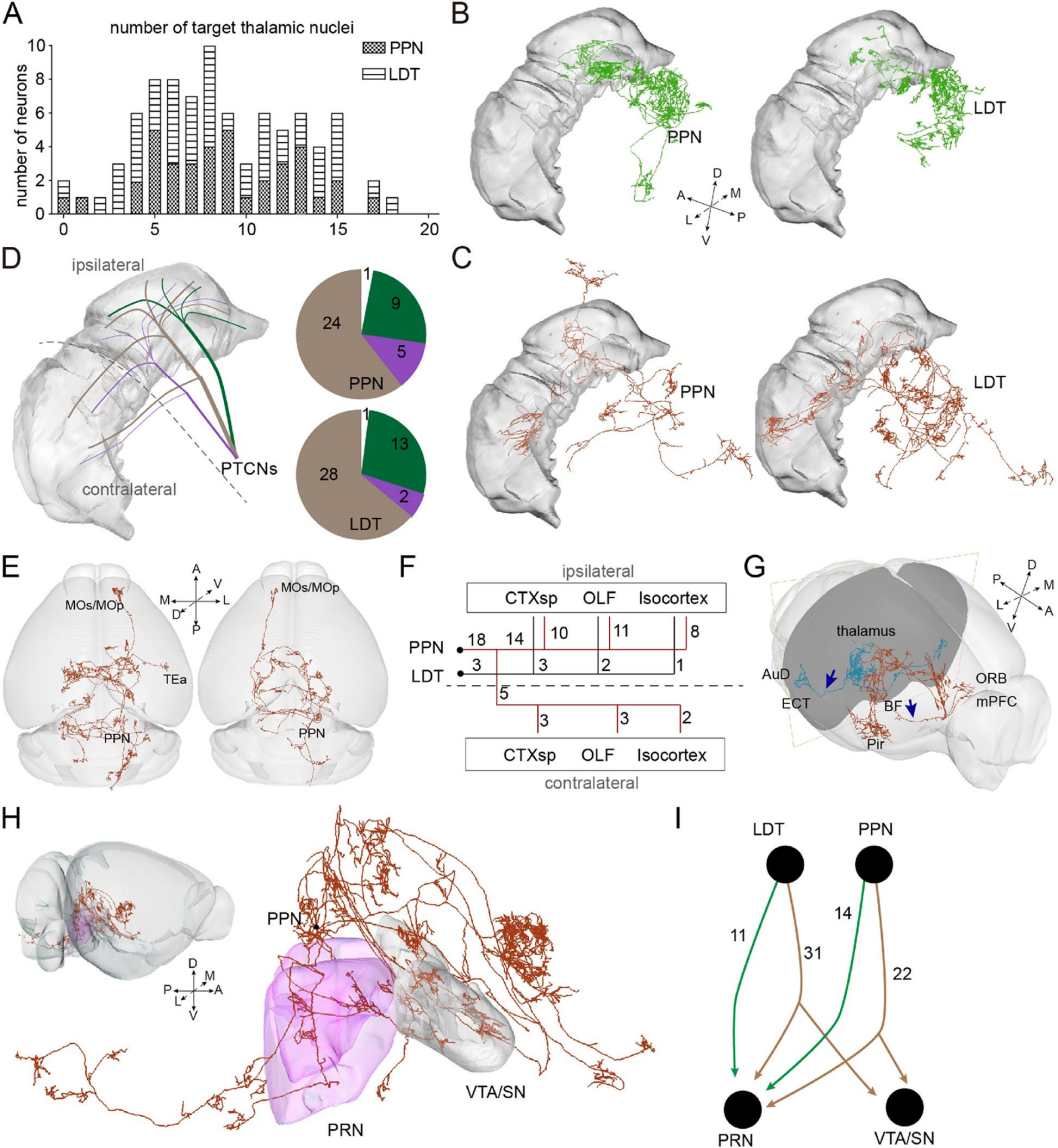
The projection logic in ascending and descending circuits. **A** The number of thalamic nuclei innervated by individual PTCN. **B** Typical neurons only innervate the ipsilateral thalamus. **C** Typical neurons prefer the contralateral thalamus. **D** Schematic diagram and number of three patterns of PTCNs innervating the thalamus. **E** 3D view of typical neurons projecting to the ipsilateral (left) and contralateral (right) cortex. **F** The targets of cortical-projecting neurons. **G** Two separated pathways project to rostral and lateral cortex. **H** 3D view of typical neurons projecting to the PRN and VTA/SN simultaneously. **I** The number of reconstructed PTCNs projecting to the PRN and VTA/SN. For the details of abbreviations for brain regions see the “Abbreviations” section. For detailed statistics of thalamic terminals, see Additional file 3: Table 2

The morphology of individual neurons suggests that most PTCNs (81/83) send abundant axons to the thalamus (Fig. 2A). As the main targets of PTCNs, we quantified the proportion of thalamic terminals in individual neurons and found that this exhibited considerable heterogeneity (PPN, 0–84.76%; LDT, 0–49.56%) (Additional file 1: Fig. S5A). In agreement with our previous study [9], the PPN neurons had richer thalamic collaterals on average, but no significant difference compared with the LDT neurons (average thalamic terminals: PPN, 21.29%; LDT, 14.98%). To explore cholinergic projection logic in the thalamus, we divided the bilateral thalamus into 22 subregions according to Allen CCFv3 and counted the terminals of individual neurons (Additional file 1: Fig. S5B). Consistent with the output atlas [9], both the PPN and LDT cholinergic neurons showed extensive collateralization in the bilateral thalamus, especially in the anterior, ventral, and medial areas. Single PTCNs tend to innervate multiple thalamic nuclei, and more than 80% simultaneously target at least five thalamic nuclei. One of these projected onto 18 thalamic nuclei (Fig. 4A). We then quantified the proportion of terminals in different thalamic nuclei and found that the nuclei contained more than 25% of thalamic terminals as the main thalamic targets (Additional file 1: Fig. S5C). Consistent with the output atlas [9] and synaptic distribution in rats [31], individual PPN neurons prefer thalamic motor nuclei such as the ventral group of the dorsal thalamus (VENT), while LDT neurons prefer thalamic limbic nuclei such as the anterior group of the dorsal thalamus (ATN). Individual PTCNs showed different axonal selections in the bilateral thalamus (Fig. 4B, C). To verify the organization of the cholinergic axons in the bilateral thalamus, we quantified the proportion of terminals in the ipsilateral thalamus (Additional file 1: Fig. S5D). As shown in Fig. 4D, the PPN and LDT cholinergic neurons had similar projection patterns in the bilateral thalamus. Most thalamus-projecting PTCNs synchronously targeted the bilateral thalamus; approximately one-quarter (PPN, 9/38 neurons; LDT, 13/43 neurons) of them only innervated the ipsilateral thalamic nuclei (Fig. 4D; Additional file 1: Fig. S5D). We also found that a few PTCNs (PPN, 5/38 neurons; LDT, 2/43 neurons) projected richer collaterals to the contralateral thalamus, which may prefer the contralateral thalamus and contribute to information integration in the bilateral areas. These results suggest three distinct patterns of dominance from the PTCNs to the bilateral thalamus (Fig. 4D).

The cortex is the advanced center of the nervous system and the thalamus is the main source of subcortical information in the cortical regions [33, 34]. Previous studies have shown that the activity of PTCNs influences the function of cortical neurons via indirect circuits [35, 36], little is known about their direct pathways. Our results revealed stable projections from the PTCNs to different cortices in both hemispheres (Fig. 4E), most of which originated from the PPN (Fig. 4F). We then analyzed the cortical projection neurons and found two separate pathways extending to the cortex. Some axons stretched through the basal forebrain and projected to the rostral cortex, whereas others passed through the lateral thalamus and reached the lateral cortex, including the perirhinal areas (Fig. 4G). We found that the PTCNs targeting the cortex also had rich collaterals in the thalamic nuclei (Fig. 2A).

In the descending circuits, the axons of the PTCNs were widely distributed in the pons and medulla, with some extending to the spinal cord. Previous studies have shown that activating the PPN cholinergic neurons ascending to the VTA/SN [19, 37] and descending to the lower brainstem, such as the pontine reticular nucleus (PRN) [11], has opposite effects on locomotion. We analyzed the relationship between these two functionally different circuits using a single-cell morphology atlas. As shown in Fig. 4H and I, most of the reconstructed PPN cholinergic neurons (36/39 neurons) targeted the PRN and more than half (22/36 neurons) co-projected onto the VTA/SN. We did not find a single neuron innervating the VTA/SN that did not co-project to the PRN. We also investigated cholinergic circuits from the LDT to the VTA/SN and PRN. Similar to the PPN, the majority of LDT neurons (42/44 neurons) projected to the PRN, and approximately three-quarters (31/42) co-projected to the VTA/SN. These findings indicate that most individual cholinergic axons from PTCNs to the VTA/SN simultaneously project to the PRN, whereas a minor group of PTCNs solely innervates the PRN.

## Discussion

PTCNs project to multiple regions of the ascending and descending circuits and participate in various functions. In this study, we present the detailed morphology of PTCNs in the entire brain and reveal their projection logic at the single-cell level. Individual PTCNs participate in in the ascending and descending circuits simultaneously and can be divided into four groups with different projection patterns. In the ascending circuits, individual PTCNs innervate the bilateral thalamus in three distinct patterns according to the axonal distribution in the bilateral thalamus. PTCNs project directly into the cortex via two separate pathways.

### The complex morphology of PTCNs

Projecting neurons contribute to multiple functions via complex connections with various nuclei. However, little is known about how these neurons are organized, such as whether neurons innervating different regions belong to the same or separate groups. Studying the morphology of individual neurons allows us to investigate projection patterns at the single-cell level. For example, individual cholinergic neurons in the basal forebrain can be grouped into different types according to the diverse projection preferences of the olfactory bulb, cortex, and hippocampus [24]. Similarly, using projection logic, we grouped the individual PTCNs into four types with different projection preferences. Single PTCNs contain abundant axons that simultaneously innervate tens of nuclei in the thalamus, midbrain, and pons. Our results indicated that PTCNs projecting to the three major axonal trajectories [11] were mainly from the same groups rather than segregated clusters, which may explain how the pontine-tegmental cholinergic system participates in a variety of functions with a finite number of neurons.

The complexity of PTCNs can be understood in two ways. On the one hand, most reconstructed neurons projected to ascending and descending circuits synchronously, which was consistent with previous views [25]. The upper brainstem is an important area of interaction for the transmission of ascending and descending information. PTCNs exhibit different projection patterns from other neuromodulator neurons, including serotonergic neurons in the DR [7] and DA neurons in the VTA [4], which mainly project to ascending circuits with a few axons projecting to descending circuits. However, a few of them have synchronous collaterals in the ascending and descending circuits [7]. Glutamatergic neurons in the PPN [38] and VTA [39] also have rich axons in both the ascending and descending circuits, but their single-cell projection patterns remain unknown. Moreover, the activation of PPN cholinergic neurons plays an opposite role in locomotion via the ascending and descending circuits [11], which means that these two circuits may belong to different groups. However, we found that all the reconstructed VTA/SN-projecting PPN cholinergic neurons had collaterals in the PRN. These results show that the opposite functions of the two cholinergic circuits may originate from different neurons or different responses of downstream neurons to the same neurons. Hence, PTCNs, which modulate many ascending and descending circuits, mainly originate from the same group of neurons.

On the other hand, we found that some neurons had richer collaterals in contralateral regions, which may have delivered more information to the contralateral hemisphere. The axial selection of ipsilateral and/or contralateral targets is crucial for integrating bilateral information and coordinated movements. PTCNs decrease significantly in patients with Parkinson’s disease (PD); characterized by abnormalities of movement, including tremors and difficulties with gait and balance [40]. The PPN is an important clinical target for deep brain stimulation in patients with PD [41], emphasizing the role that PTCNs may play in somatic homeostatic regulation by projecting abundant axons to bilateral hemispheres.

### Projection logic of cholinergic axons ascending to the thalamus

The thalamus is an important relay station for sensorimotor information in mammals and acts as the main pathway for the pontine-tegmental cholinergic system to deliver information in ascending circuits. As the main source of acetylcholine for the thalamus, PTCNs project to the thalamus and form synaptic connections [9, 31]. In this study, we uncovered the logic of projection of the PTCNs to the thalamus at the single-cell level. Consistent with previous studies [42], most PTCNs directly innervate the thalamic nuclei. Individual PTCNs synchronously target multiple thalamic nuclei. We found that more than 80% of the reconstructed PTCNs innervated at least five thalamic nuclei, and some of them simultaneously projected to 18 nuclei in the bilateral thalamus. This indicates that the regulation of PTCNs in the thalamus is mainly diffuse, rather than concentrated in a few regions. The thalamic nuclei receive information from the whole brain and deliver it to diverse cortical areas [33, 34]. We speculate that individual PTCNs send complex information to various thalamic nuclei, which sort the information and then deliver it to specific cortical fields. The diverse functions of thalamic circuits projected from the PTCNs [13–15] may originate from the different responses of different thalamic nuclei.

Moreover, we decoded three projection patterns from PTCNs with different preferences in the ipsilateral and/or contralateral thalamus. There may be three different modes of information transmission from the pontine-tegmental cholinergic system to the thalamus for further information progress. Specifically, a large number of PTCNs targeted the bilateral thalamus and some preferred contralateral areas. These may play important roles in coordinated movement and contribute to some movement disorders diseases, such as PD [40]; the cholinergic circuits in the thalamus of these patients exhibit abnormal activity [14].

### Morphological differences between the PPN and LDT cholinergic neurons

PPN and LDT cholinergic neurons are two components of the pontine-tegmental cholinergic system that have diverse characteristics with regard to connections and functions [9, 37]. They connect with similar regions with different preferences [9, 11, 31]. In this study, we demonstrated that the PPN and LDT cholinergic neurons have similar projection patterns with different preferences at the single-cell level. Comparatively, the PPN projected more divergent cholinergic axons to the ascending and descending circuits, whereas LDT neurons were more convergent. Various studies have suggested that the PPN and LDT cholinergic neurons target the same regions with different functions, such as the VTA [37] and SN [19]. The functional difference is related to the connectional difference between the two cholinergic nuclei: PPN neurons prefer motor nuclei, whereas LDT neurons prefer limbic nuclei [9, 11, 31]. Furthermore, single PTCNs have abundant and diverse co-projection axons that may contribute to functional differences. The thalamus is one of the main targets of PTCNs, and both individual PPN and LDT cholinergic neurons have rich collaterals in different thalamic nuclei with similar projection logic; however, they also show some differences [9, 31]. On the one hand, the PPN neurons had richer thalamic collaterals on average, and we found that some projected more than 50% of their axons to the thalamus. In contrast, the two groups of cholinergic neurons had different preferences for thalamic nuclei: PPN neurons preferred the thalamic motor nuclei, while LDT neurons preferred the limbic nuclei. In addition, the dendrites of the LDT cholinergic neurons had richer branches and longer dendrites than those of the PPN. As neuronal portals receive information, dendrites influence the number and identity of presynaptic inputs [43], which implies that LDT neurons may receive more afferent information.

## Conclusions

Our study elucidated the single-cell morphology of PTCNs on a brain-wide scale and decoded the projection logic of the whole brain and specific circuits. We revealed that individual PTCNs send abundant axons in multiple nuclei in the ascending and descending circuits synchronously and can be grouped into four types with different projection patterns. Furthermore, we uncovered three different projection patterns from the PTCNs to the bilateral thalamus, and two separate pathways to the cortical areas. Our study mapped the axonal projections of the pontine-tegmental cholinergic system; thus providing researchers with a better understanding of its projection logic.

## Methods

### Animals

Adult (2–4 months) ChAT-ires-Cre mice were used in this study. ChAT-Cre transgenic mice (stock No: 018957) were purchased from Jackson Laboratory. The mice were kept under a condition of a 12-h light/dark cycle with food and water ad libitum. All animal experiments were approved by the Animal Care and Use Committee of Huazhong University of Science and Technology.

### Tracer information

For sparse labeling, we employed the CSSP-YFP (5.2 × 10^12^ VG/mL) packed by Brain Case (Brain Case Co., Ltd., Shenzhen, China). CSSP-YFP virus is produced by co-packaging the rAAV-EF1α-DIO-Flp plasmid and rAAV-FDIO-EYFP plasmid with a ratio of 1:20,000 in the single rAAV production step [28].

### Surgery and viral injection

Before virus injection, we anesthetized the mice with mixed anesthetics (2% chloral hydrate and 10% ethyl urethane dissolved in 0.9% NaCl saline) according to their weight (0.1 ml/10 g). The brain of anesthetized mice was fixed with a stereotaxic holder to adjust the position of the skulls. Then a cranial drill (~ 0.5 mm diameter) was employed to uncover the skulls above the targets. For sparse labeling, we injected 100 nl CSSP-YFP into the unilateral PPN or LDT.

### Histology and immunostaining

The histological operations followed previous studies [33]. Shortly, four weeks after AAV injection, anesthetized mice were perfused with 0.01 M PBS (Sigma-Aldrich, USA), followed by 2.5% sucrose and 4% paraformaldehyde (PFA, Sigma-Aldrich, USA) in 0.01 M PBS. Then, the brains were removed and post-fixed in 4% PFA solution overnight.

For immunofluorescent staining, some samples were sectioned in 50 μm coronal slices with the vibrating slicer (Leica 1200S). All sections containing PPN or LDT were selected to characterize the labeled neurons in inject site. These sections were blocked with 0.01 M PBS containing 5% (wt/vol) bovine serum albumin (BSA) and 0.3% Triton X-100 for 1 h at 37 °C. Then the sections were incubated with the primary antibodies (12h at 4 °C): anti-ChAT (1:500, goat, Sigma-Aldrich, AB144P). Then the sections were washed in PBS five times at room temperature. Next, these sections were incubated with the fluorophore-conjugated secondary antibody (1:500, Abcam: Alexa-Fluor 647, donkey anti-goat) for 2 h at room temperature. After rinsing with PBS, DAPI (1 ng/mL) was performed on stained sections for 5 min, and sections were finally mounted after washing. We acquired the stained information of sections with the confocal microscope (LSM 710, Zeiss, Jena, Germany).

### Imaging and 3D visualization

For whole-brain imaging, virus-labeled samples were dehydrated with alcohol and embedded with resin. Then the whole brain datasets were acquired with the fMOST system. In short, we fixed the embedded sample on the base and acquired the image of the top surface with two fluorescent channels; the imaged tissue was subsequently removed by diamond wheel. Thus, we obtained the continuous whole-brain dataset layer by layer with high resolution (0.32 μm × 0.32 μm × 1 μm).

For 3D visualization and statistical analysis of whole-brain datasets, we registered the whole-brain datasets to the Allen CCFv3 [30]. The methods of registration have been described previously [44]. Briefly, we employed image preprocessing to correct uneven illumination and then remove background noise. The down-sampling data (the voxel resolution of 10 μm × 10 μm × 10 μm) was uploaded into Amira software (v6.1.1, FEI, Mérignac Cedex, France) to distinguish and extract regional features of anatomical invariants, including the outline of the brain, the ventricles, and the corpus striatum, etc. Next, the gray-level-based registration algorithm (SyN) was employed to register the extracted features. Basic operations including extraction of areas of interest, resampling, and maximum projection performed via Amira software and Fiji (NIH).

### Morphological reconstruction of single neurons

83 neurons were reconstructed from six brains. For single-cell morphological analysis, we reconstructed the morphology of sparsely labeled neurons with semi-automatic methods following previous studies [29]. Briefly, we acquired the spatial coordinates of labeled somas in high-resolution data and transformed the data format of GFP-labeled data from TIFF to TDI type via Amira first.

Then the data block containing the given soma was loaded into GTree software and we assigned the soma as the initial point and marked all its axons with unfinished tags. Next, we selected one uncompleted fiber and traced it in the next block with automatic tracing. Then we checked the traced fiber and marked its branches with unfinished tags. We repeated the above procedure until the selected fiber was finished and then we reconstructed the remaining unfinished axons until all the axons were achieved. The reconstructed neurons were checked back-to-back by three persons. The tracing results were saved in SWC format. Meanwhile, we registered the PI-labeled data and the corresponding tracing results to the reference atlas with the methods mentioned above. Considering the distribution of axons and synaptic connection does not always correlate [31], we counted the terminal branches of reconstructed neurons to represent the connection between single cholinergic neurons and their targets.

### Statistical information

To distinguish the axons of passage vs. terminal innervations, we take the ended axons as terminal axons while others as passage axons. For terminals quantification, the reconstructed neurons carried spatial information of all nodes that we could calculate the ended nodes based on registered single neurons to obtain the terminals in different regions. In addition, we employed the Amira software to quantify the length of dendrites and axons of individual neurons. Statistical graphs were generated using GraphPad Prism v.8.02 and Microsoft Excel (Office 2020). We employed GraphPad Prism v. 8.02 for the significance test, Neurolucida360 software for the polar histogram, and MATLAB (2017a) for the Sholl Analysis. We conducted one-way ANOVA followed by Tukey’s post hoc tests to compare the difference among four projections patterns of same nuclei while two-tailed t-tests to compare the difference between the PPN and LDT. The confidence level was set to 0.05 (*P* value) and all results were presented as mean ± SEM.

## Supplementary Information

**Additional file 1:** **Fig. S1.** Sparse labeling of cholinergic neurons.Sparsely labeled 3D continuous datasets.Typicalinject site of the PPN and LDT.Labeled fibers rangefrom the cortical areas to the spinal cord. Scale bar,1000μm;200μm;100μm. **Fig. S2.** 3D view of all 83reconstructed cholinergic neurons. Pentagons presented neurons had richer axonbranches in the contralateral hemisphere. Red triangle pointed neuronrestricted its fibers in the ipsilateral thalamus. Green triangle-pointedneurons sent richer fibers to the contralateral thalamus. **Fig. S3.** PFL-projectionneurons and the soma of reconstructed neurons.3D view of three neurons projecting to thePFL.3Dview of all reconstructed soma. **Fig. S4.**Morphology and polar analysis of 83 reconstructed neurons. **Fig. S5.** Quantitative analysisof axons of thalamic projection PTCNs.the proportion of thalamic terminals in individualneurons. Two-sided t-tests.The distribution of axonal terminals in the thalamus of singleneurons. Each column displayed one neuron. Boxes in different colors explainedthe number of terminals of a single neuron in different brain regions.The number of mainthalamic targets of individual PTCNs.The ratio ofterminals in the ipsilateral thalamus of single neurons. Green dots showedneurons confined their axons in the ipsilateral thalamus. Purple-filled dotsrepresent neurons that had richer axons in the contralateral thalamus. Bluedots represent neurons that did not target the thalamus. For the details of abbreviations for brainregions see Nomenclature and abbreviations. For detailed statistics of thalamicterminals, see Additional file 3: Table 2.

**Additional file 2:** **Table 1.** Quantification of reconstruction neurons.

**Additional file 3:** **Table 2.** Quantification of axonal terminals in the thalamus.

## Data Availability

All data generated or analyzed during this study are included in this published article and its supplementary information files. Supplemental Information includes five figures and two tables and can be found additional files. The TB-sized raw data of sparse-labeling samples and 3D data of reconstructed neurons can be accessed at http://atlas.brainsmatics.org/a/zhao2206.

## Abbreviations

PPN: Pedunculopontine nucleus
LDT: Laterodorsal tegmental nucleus
PTCNs: Pontine-tegmental cholinergic neurons
CTXpl: Cortical plate
CTXsp: Cortical subplate
OLF: Olfactory areas
ORB: Orbital area
MOs: Secondary motor area
MOp: Primary motor area
mPFC: Medial prefrontal cortex
ECT: Ectorhinal area
PERI: Perirhinal area
TEa: Temporal association areas
AuD: Auditory areas
STR: Striatum
PAL: Pallidum
BF: Basal forebrain
RT: Reticular nucleus of the thalamus
VENT: Ventral group of the dorsal thalamus
GENd: Geniculate group, dorsal thalamus
MED: Medial group of the dorsal thalamus
LAT: Lateral group of the dorsal thalamus
ILM: Intralaminar nuclei of the dorsal thalamus
GENv: Geniculate group, ventral thalamus
EPI: Epithalamus
ATN: Anterior group of the dorsal thalamus
DORpm: Thalamus, polymodal association cortex related
DORsm: Thalamus, sensory-motor cortex related
PVR: Periventricular region
PVZ: Periventricular zone
LZ: Hypothalamic lateral zone
MEZ: Hypothalamic medial zone
MBmot: Midbrain, motor related
MBsen: Midbrain, sensory related
MBsta: Midbrain, behavioral state related
VTA: Ventral tegmental area
SN: Substantia nigra
P-mot: Pons, motor related
P-sat: Pons, behavioral state related
P-sen: Pons, sensory related
PRN: Pontine reticular nucleus
CB: Cerebellum
PFL: Paraflocculus
MY: Medulla
MY-sen: Medulla, sensory related
MY-sat: Medulla, behavioral state related
MY-mot: Medulla, motor related
