# Supplementary material for: Upper brainstem cholinergic neurons project to ascending and descending circuits
Source: BMC Biol. 2023 Jun 6;21:135. doi: 10.1186/s12915-023-01625-y (PMC10245412; doi:10.1186/s12915-023-01625-y)
Supplement: Supplementary file 1 — Additional file 1: Fig. S1. Sparse labeling of cholinergic neurons.Sparsely labeled 3D continuous datasets.Typicalinject site of the PPN and LDT.Labeled fibers rangefrom the cortical areas to the spinal cord. Scale bar,1000μm;200μm;100μm. Fig. S2. 3D view of all 83reconstructed cholinergic neurons. Pentagons presented neurons had richer axonbranches in the contralateral hemisphere. Red triangle pointed neuronrestricted its fibers in the ipsilateral thalamus. Green triangle-pointedneurons sent richer fibers to the contralateral thalamus. Fig. S3. PFL-projectionneurons and the soma of reconstructed neurons.3D view of three neurons projecting to thePFL.3Dview of all reconstructed soma. Fig. S4.Morphology and polar analysis of 83 reconstructed neurons. Fig. S5. Quantitative analysisof axons of thalamic projection PTCNs.the proportion of thalamic terminals in individualneurons. Two-sided t-tests.The distribution of axonal terminals in the thalamus of singleneurons. Each column displayed one neuron. Boxes in different colors explainedthe number of terminals of a single neuron in different brain regions.The number of mainthalamic targets of individual PTCNs.The ratio ofterminals in the ipsilateral thalamus of single neurons. Green dots showedneurons confined their axons in the ipsilateral thalamus. Purple-filled dotsrepresent neurons that had richer axons in the contralateral thalamus. Bluedots represent neurons that did not target the thalamus. For the details of abbreviations for brainregions see Nomenclature and abbreviations. For detailed statistics of thalamicterminals, see Additional file 3: Table 2. [file 12915_2023_1625_MOESM1_ESM.docx]

# Upper Brainstem Cholinergic Neurons Project to Ascending and Descending Circuits

Peilin Zhao^1,4 #^, Tao Jiang^3#^, Huading Wang^1^, Xueyan Jia^3^, Anan Li^1, 3^, Hui Gong^1, 3^, Xiangning Li^1, 2, 3^ *

^1^ Britton Chance Center for Biomedical Photonics, Wuhan National Laboratory for Optoelectronics, MoE Key Laboratory for Biomedical Photonics, Huazhong University of Science and Technology, Wuhan 430074, China

^2^ Key Laboratory of Biomedical Engineering of Hainan Province, School of Biomedical Engineering, Hainan University, Haikou 570228, China

^3^ HUST-Suzhou Institute for Brainsmatics, JITRI, Suzhou 215123, China

^4^Institute of neurological diseases, North Sichuan Medical University, Nanchong 637100, China

## *Correspondence*

* Xiangning Li, [lixiangning@mail.hust.edu.cn](mailto:lixiangning@mail.hust.edu.cn)

### Supplementary Figures


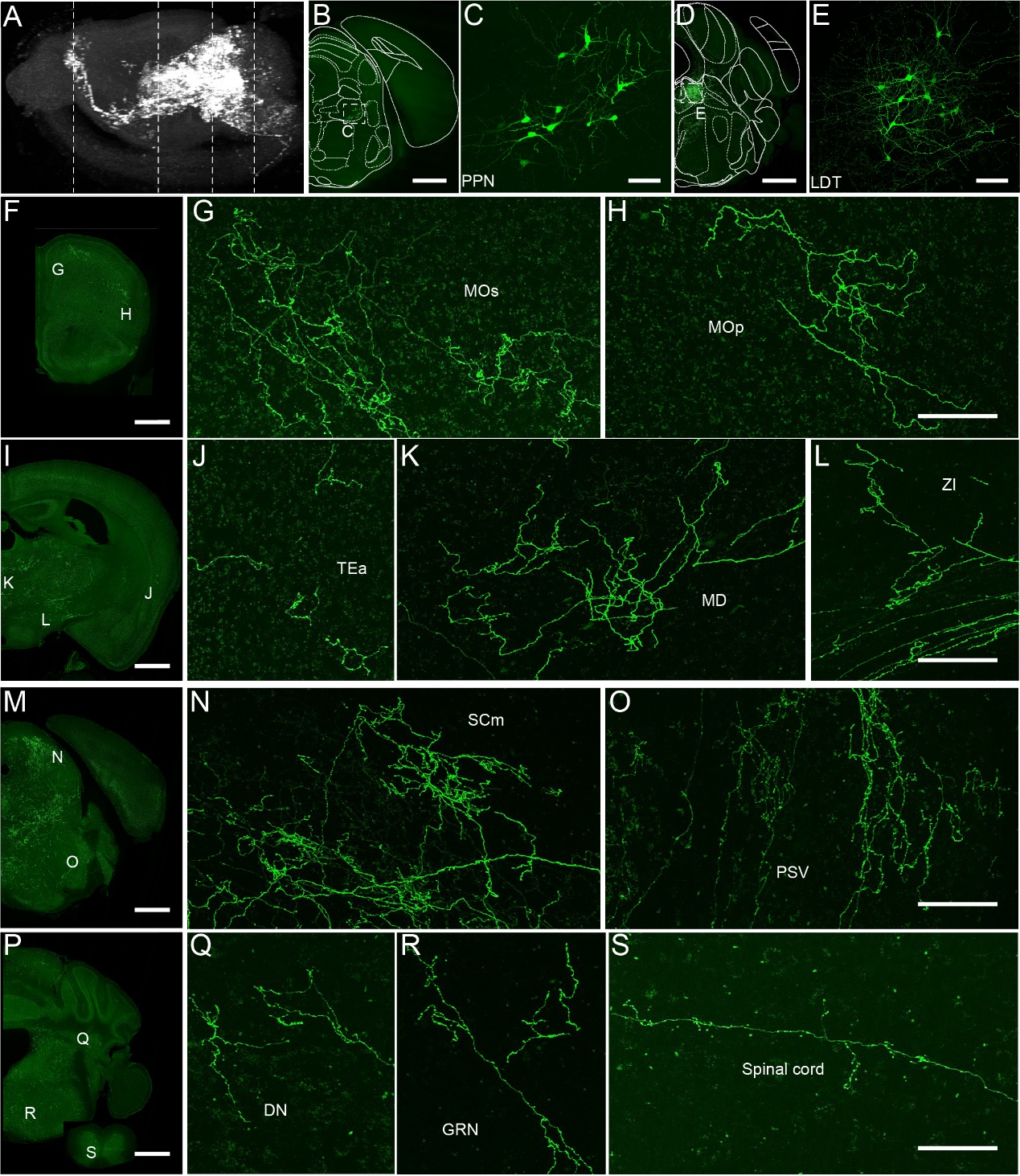


**Figure S1 Sparse labeling of cholinergic neurons.** (A) Sparsely labeled 3D continuous datasets. (B-E) Typical inject site of the PPN and LDT. (F-S) Labeled fibers range from the cortical areas to the spinal cord. Scale bar, (B, D, F, I, M, P) 1000μm; (C, E) 200μm; (G, H, J, K, L, N, O, Q, R, S) 100μm.


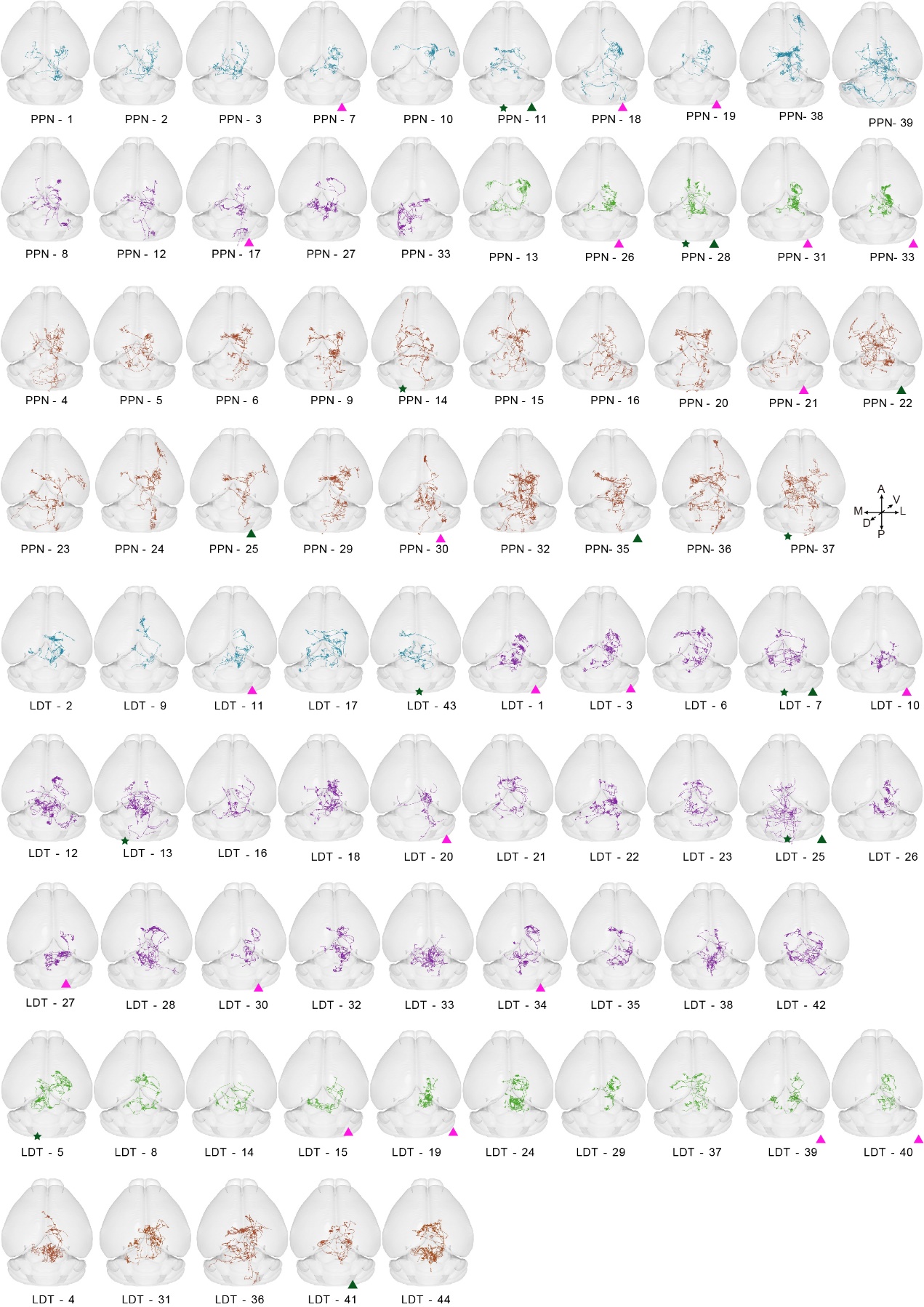


**Figure S2** **3D view of all 83 reconstructed cholinergic neurons.** Pentagons presented neurons had richer axon branches in the contralateral hemisphere. Red triangle pointed neuron restricted its fibers in the ipsilateral thalamus. Green triangle-pointed neurons sent richer fibers to the contralateral thalamus.


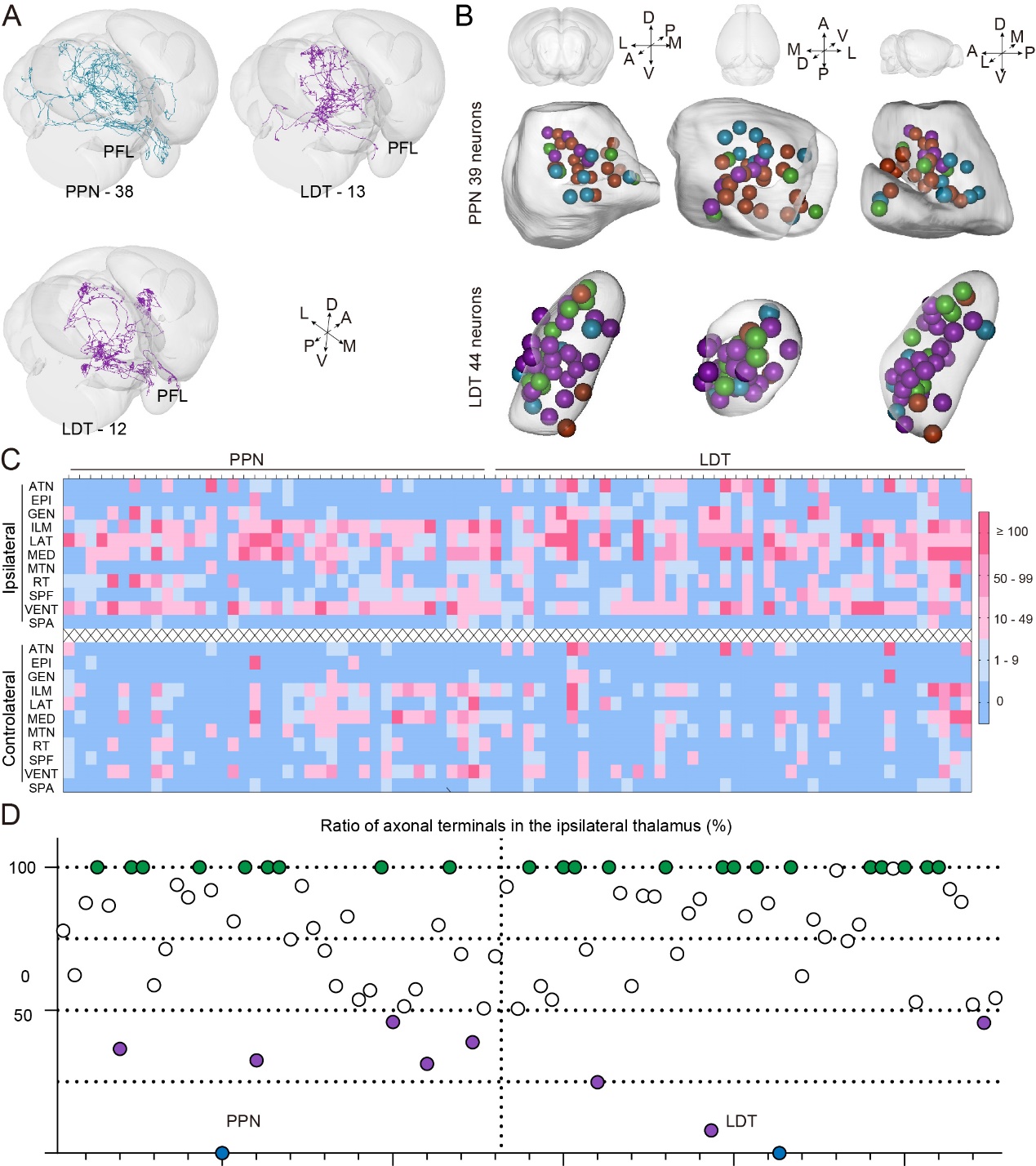


**Figure S3** **PFL-projection neurons and the soma of reconstructed neurons.** (A) 3D view of three neurons projecting to the PFL. (B) 3D view of all reconstructed soma.


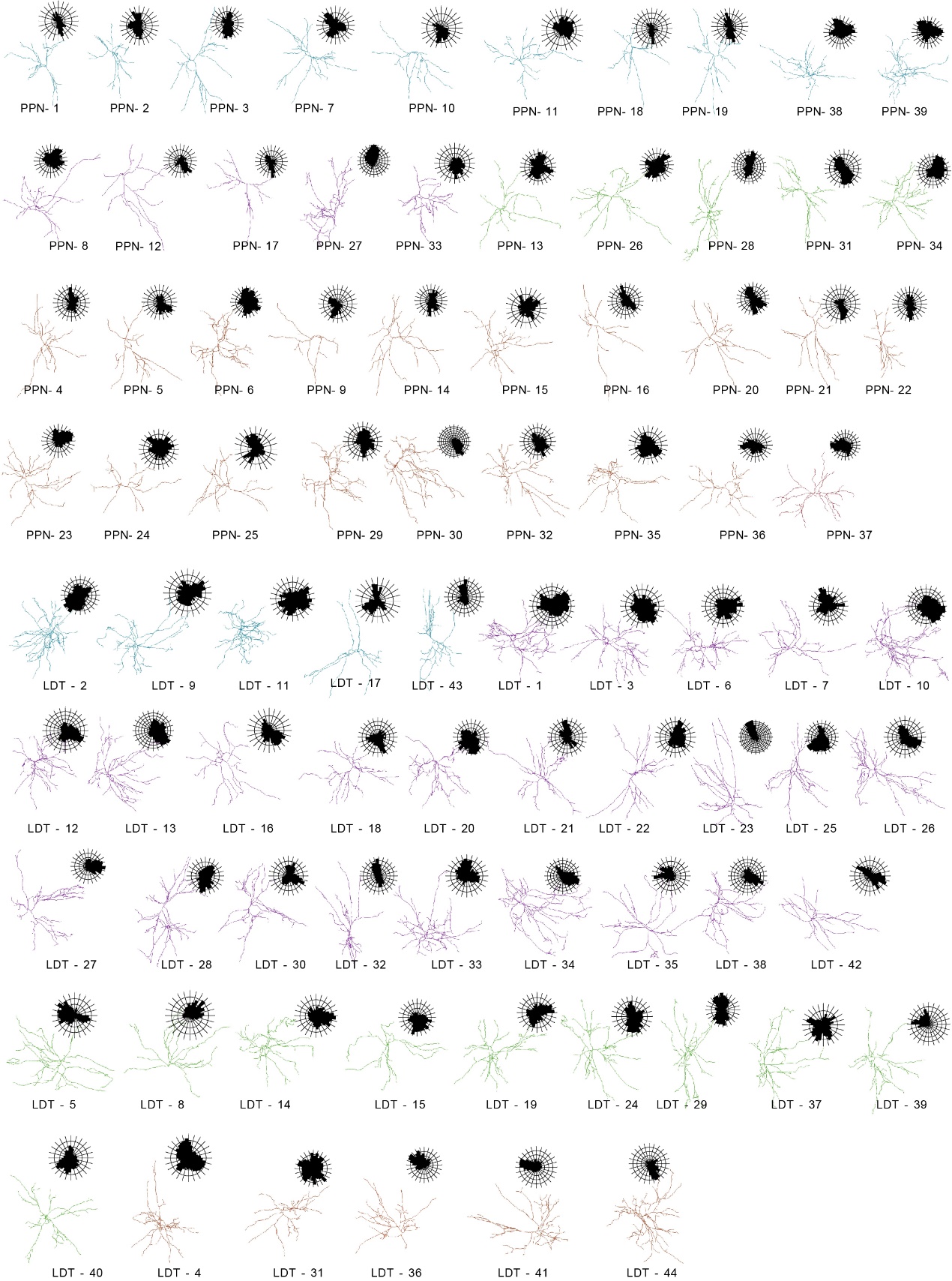


**Figure S4** **Morphology and polar analysis of 83 reconstructed neurons.**


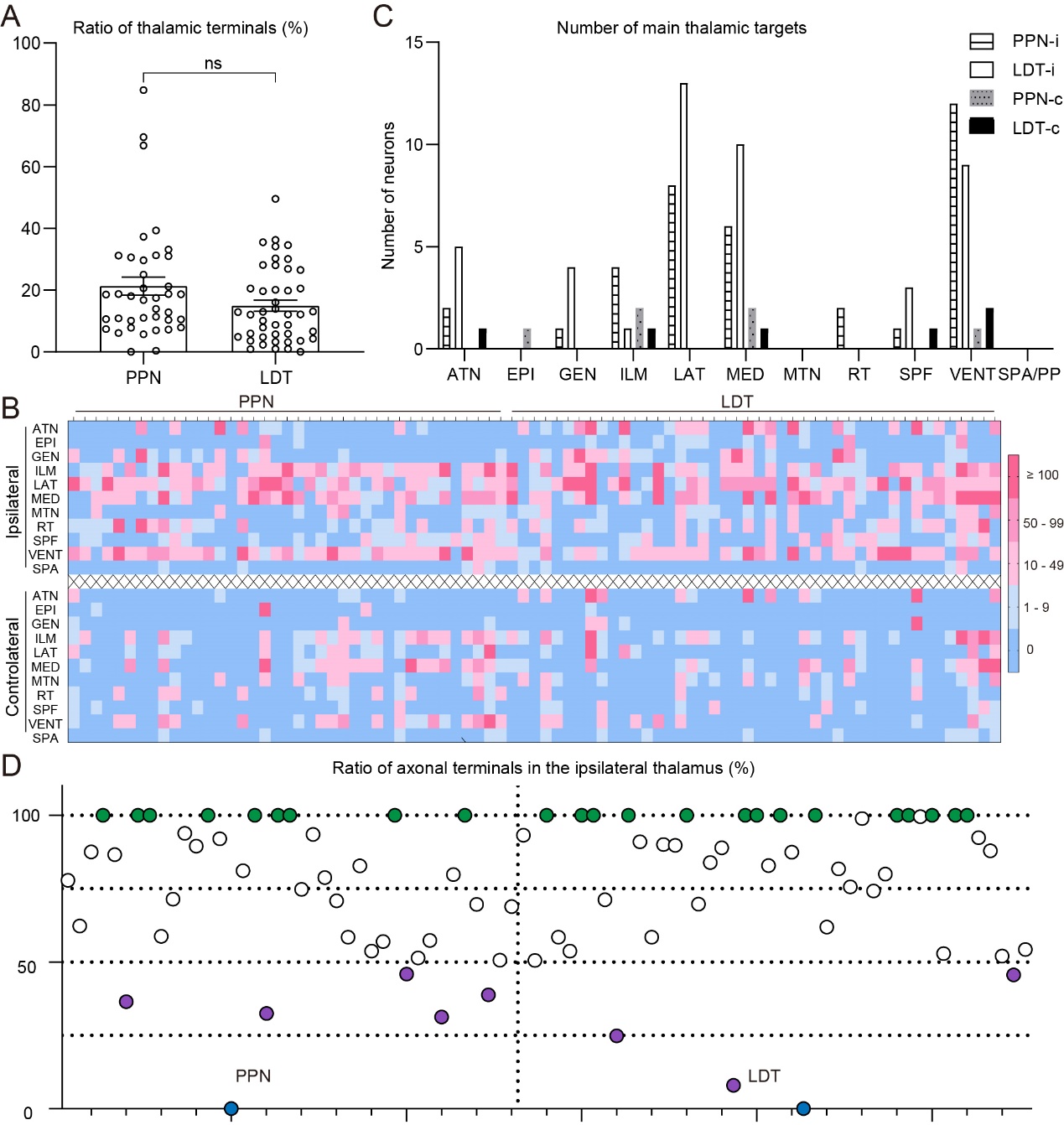


**Figure S5 Quantitative analysis of axons of thalamic projection PTCNs.** (A) the proportion of thalamic terminals in individual neurons. Two-sided t-tests. (B) The distribution of axonal terminals in the thalamus of single neurons. Each column displayed one neuron. Boxes in different colors explained the number of terminals of a single neuron in different brain regions. (C)The number of main thalamic targets of individual PTCNs. (D) The ratio of terminals in the ipsilateral thalamus of single neurons. Green dots showed neurons confined their axons in the ipsilateral thalamus. Purple-filled dots represent neurons that had richer axons in the contralateral thalamus. Blue dots represent neurons that did not target the thalamus. For the details of abbreviations for brain regions see Nomenclature and abbreviations. For detailed statistics of thalamic terminals, see Additional file 3: Table 2.
